# Environmental tobacco smoke (ETS) and hyperlipidemia modified by perceived work stress

**DOI:** 10.1371/journal.pone.0227348

**Published:** 2020-01-16

**Authors:** Ping-Yi Lin, Jong-Yi Wang, Pochang Tseng, Dann-Pyng Shih, Ching-Lan Yang, Wen-Miin Liang, Hsien-Wen Kuo

**Affiliations:** 1 Transplant Medicine & Surgery Research Centre, Changhua Christian Hospital, Changhua, Taiwan; 2 Department of Medical Research, China Medical University Hospital, Taichung, Taiwan; 3 Department of Nursing, Da-Yeh University, Changhua, Taiwan; 4 Department of Health Services Administration, China Medical University, Taichung, Taiwan; 5 Health Promotion Administration, Ministry of Health and Welfare, Taipei, Taiwan; 6 Institute of Environmental and Occupational Health Sciences, National Yang Ming University, Taipei, Taiwan; 7 International Medical Department, Changhua Christian Hospital, Changhua, Taiwan; 8 Department of Public Health, China Medical University, Taichung, Taiwan; 9 Department of Occupational Diseases, Far Eastern Memorial Hospital, New Taipei city, Taiwan; 10 School of Public Health, National Defense Medical Center, Taipei, Taiwan; University of Calfornia San Francisco, UNITED STATES

## Abstract

**Background:**

Accumulating evidence has shown that exposure to environmental tobacco smoke (ETS) is associated with cardiovascular diseases (CVDs) However, few studies have assessed both exposure to ETS and high-perceived work stress on hyperlipidemia. The aim of the present study is to assess the interaction effect of ETS exposure and high-perceived work stress on the risk of hyperlipidemia.

**Methods:**

A total of 11,875 middle-aged civil servants from 647 registered institutions employed by the Taiwan government were randomly selected using multistage stratified cluster sampling based on proportional probabilistic sampling. Each participant anonymously and independently filled out a web-based questionnaire and informed consent.

**Results:**

The prevalence of hyperlipidemia in middle-aged civil servants diagnosed by physicians was 11.5% for men and 6.1% for women. Hyperlipidemia was significantly associated with smoking, alcohol consumption, betel nut chewing, weight gain and perceived work stress. In both the obesity and smoking groups, there were consistent interaction effects of ETS exposure and perceived work stress on hyperlipidemia for middle-aged civil servants. Non-obese and non-smoking groups were more at risk for hyperlipidemia from exposure to both ETS and high-perceived work stress.

**Conclusion:**

There is an interaction effect of ETS exposure and high-perceived work stress on hyperlipidemia, regardless of obesity and smoking. It is crucial to immediately reduce ETS exposure and stressful work by enforcing smoke-free policies and reducing pressure for civil servants.

## Introduction

Since smoke-free legislation was implemented in Taiwan in 2009, the smoking rates incrementally decreased by 6.5% in men and 0.4% in women between 2005 and 2011[1]. The prevalence rates of smokers who reported quit attempts increased from 30.2% before the legislation to 51.7% after the legislation [2]. Similarly, evidence from previous studies from other countries show a decline in the prevalence of environmental tobacco smoke (ETS) exposure in the workplace and at home after implementing a smoke-free legislation, from 28.5% in July 2008 to 7.3% in March 2009 for the workplace and from 36.8% to 21.3% during the same period at home [3]. ETS, a widespread environmental toxicant, is a known risk factor for cardiovascular diseases (CVDs), which may be caused by atherosclerosis or dyslipidemia.

Cigarette smoking has been recognized as an independent and preventable risk factor for atherosclerosis and CVDs. Public health intervention programs have helped substantially decrease smoking prevalence, but the CVDs attributed to ETS exposure are by no means a thing of the past. Besides aging, ethnic group, lower income, and lower educational levels, factors related to weight gain and an increased risk of CVDs are poor access to public health services [4, 5] and unhealthy lifestyle habits including smoking, alcohol consumption, physical inactivity, disruption of sleep duration, and poor diet patterns [6]. Work-related stress has also been neglected as a contributing factor for CVDs. Some civil servants, such as police officers and firefighters, frequently have stressful workloads, shift changes, overnight work, and erratic work schedules[7–9]. Long-term exposure to crises and high-pressure situations can easily cause atherosclerosis and CVDs. Although exposure to ETS for civil servants in governmental institution has decreased, they are still frequently exposed to third-hand smoking (THS) when faced with citizens who smoke and carry smoke fumes adsorbed in their clothing or on their skin. It should be noted that few studies have found the combined effects of ETS exposure and perceived psychosocial work stress on hyperlipidemia. The effect of dyslipidemia associated with both exposure to ETS and perceived psychosocial work stress after adjusting for covariates is still inconclusive. The objective of this study is to assess the interaction effect of ETS exposure and high-perceived psychosocial work stress on the risk of hyperlipidemia.

## Methods

### Study population

This study was the first nationwide survey of civil servants workplace health and used multistage stratified random cluster sampling based on proportional probabilistic sampling (PPS). The survey was launched by Taiwan’s Health Promotion administration (HPA) to assist with initiating an intervention program to improve worker’s health and the work environment. The results are to act as a baseline to evaluate the efficacy of the intervention program for civil servants. This study obtained ethical approval by the Institutional Review Board in the China Medical University Hospital (CMUH105-REC3-091). Participants were also informed that the data would be handled confidentially. Information about this study was sent to governmental institutions to invite workers to participate in our survey. Once agreed to participate, civil servants filled out informed consent and completed a self-report online questionnaire. Our study only enrolled participants aged between 40–60 years old. The overall response rate was 35.8%. Reasons for non-response included vacation, requested time off, or having insufficient time to fill out the questionnaire, and they do not affect the validity of the data. Our results validated the reliability of the web-questionnaire with a small group of 440 civil servants who were selected and completed both an in-person and web questionnaire. We obtained findings with high consistency from two types of interviews, which indicated that our results from the web-questionnaire were reliable. In addition, we obtained consist results from both high and low response groups, and there were no significant differences in the findings from the two response groups.

### Measurement

The web-questionnaire included demographic data, anthropometric measurements (body height and weight), lifestyle habits (tobacco smoking, alcohol consumption, betel nut chewing, duration of sleep, and frequency of physical activity), and history of diseases. Our study population was categorized into smokers and never smokers. Non-smokers or former smokers were originally included in the category of smokers because the sample size of former smokers was quite small. In this study, ETS exposure was defined as living with family members who smoked (ETS at home) or working in the same office as colleagues or managers who smoked (ETS in workplace). Effort-reward imbalance (ERI) was used to assess the level of perceived psychosocial work stress [10]. ERI was calculated using three items that measured ‘effort’ and seven items that measured ‘reward’ [11]. The ERI ratio was computed using the sum of the scores for efforts as the numerator (Efforts) and the sum of the scores for rewards as the denominator (Rewards) with an ERI ratio > 1 representing high perceived psychosocial work stress in the workplace. Cronbach's alpha coefficients for each scale in the ERI model ranged from 0.65–0.88, which was consistent the Siegrist’s study (i.e. 0.61–0.91) [11].

The web-questionnaire had three questions regarding hyperlipidemia: “Have you suffered from hyperlipidemia?” (self-perceived), “Has the disease been diagnosed by physicians?” (physician diagnosis) and “Is the disease being treated and followed up by a physician?” (ongoing treatment). We used the physician’s diagnosis as the measurement for hyperlipidemia. Overweight and obesity were defined using body mass index (BMI) measured by weight and height ratio (kg/m^2^). Based on Taiwan’s HPA guideline (2012) for overweight and obesity for Taiwanese adult men and women, BMI was categorized as follows: 18.5–24 (healthy), 24.1–27.0 (overweight), 27–30 (mild obesity), 30–35 (moderate obesity), and 36 and higher (severe obesity).

### Statistical analysis

SPSS 24 was used to analyze the data. Univariate analysis was used to examine the difference between the two ERI groups on demographic information. Hyperlipidemia was associated with the following risk factors: ERI, lifestyle, overweight, obesity and levels of BMI based on univariate analysis. Multiple logistic regression adjusted for gender, age, education level, income, and marital status was used to assess ERI levels associated with hyperlipidemia for both groups of ETS exposure, obesity, and lifestyle habits. Interaction effect of ETS exposure and ERI on risks of hyperlipidemia in the non-obese and obese groups were assessed using multiple logistic regression adjusted for gender, age, education level, income, marital status, and smoking. Sensitivity analysis was used to examine the interaction effect using different measurements for hyperlipidemia. The interaction effect of ETS exposure and ERI on hyperlipidemia was measured by relative excess risk due to interaction (RERI), attributable proportion due to interaction (AP) and synergy index (S). A resulting confidence interval of RERI > 0 or AP > 0 or S > 1 indicated an additive interaction (available at website with http://www.epinet.se.).

## Results

Table 1 showed there were significant differences in demographic information between the ERI <1 and the ERI>1 group. Prevalence rates of hyperlipidemia in middle-aged civil servants diagnosed by physicians were 11.5% for men and 6.1% for women, which were lower than those with self-perceived hyperlipidemia (17.3% for men and 9.5% for women). Univariate analysis of hyperlipidemia and risk factors are shown in Table 2. The ERI >1 group had a 1.38 times greater risk of hyperlipidemia compared with the ERI<1 group. Lifestyle habits including tobacco smoking, alcohol consumption, and betel nut chewing had higher ORs (1.84, 1.67 and 2.84, respectively) for hyperlipidemia. Risk of hyperlipidemia were associated with weight gain, with a 2.94 times greater risk for obesity and a 1.75 times of being overweight. BMI levels were dose-dependently associated with the risk of hyperlipidemia.

**Table 1:** Demographic information for both ERI groups.

	1 ≤ ERI (N = 6165) n (%)	1 >ERI (N = 5612) n (%)	Total (N = 11777) n (%)	P
Gender				<0001
Men	3050(49.5)	2380(42.4)	5430(46.1)	
Women	3115(50.5)	3232(57.6)	6347(53.9)	
Age (years)				<0001
40–49	3713(60.2)	3939(70.2)	121(59.9)	
50–60	2457(39.8)	1676(29.8)	81(40.1)	
Education level				<0001
Senior high school	488(7.9)	311(5.5)	799(6.8)	
College	1558 (25.3)	1239(22.1)	2797(23.8)	
Undergraduate	2609(42.4)	2549(45.5)	5158(43.9)	
Graduate	1492(24.3)	1507(26.9)	2999(25.5)	
Marital status				0.004
Unmarried	866(14.0)	911(16.2)	1777(15.1)	
Married	5206(84.4)	4617(82.3)	9823(83.4)	
Other	95(1.5)	85(1.5)	180(1.5)	
Salary				<0001
Low	434(7.0)	378(6.7)	812(6.9)	
Moderate	4644 (75.3)	4523(80.6)	9167(77.8)	
High	1088(17.6)	711(12.7)	1799(15.3)	

**Table 2:** Univariate analysis of hyperlipidemia and risk factors.

	N = 1023 n (%)	OR (95% CI)	P
ERI			<0001
≤ 1	459(7.4)	1	
>1	564(10.0)	1.39(1.22–1.58)	
ETS			0.011
No	772(8.3)	1	
Yes	251(9.9)	1.22(1.05–1.41)	
Tobacco smoking			<0.001
No	728(7.5)	1	
Yes	295(14.1)	2.03(1.76–2.35)	
Alcohol consumption			<0001
No	933(8.2)	1	
Yes	190(11.6)	1.48(1.23–1.75)	
Betel nut chewing			0.001
No	917(8.2)	1	
Yes	106(10.4)	2.12(1.71–2.64)	
Obesity			<0.001
No	682(6.9)	1	
Yes	339(18.1)	2.94(2.41–3.58)	
Overweight			<0.001
No	321(4.9)	1	
Yes	700(13.4)	1.75(1.27–2.41)	
BMI			
<18.4	14(3.4)	1	
18.5–24	307(5.0)	1.51(0.87–2.60)	0.140
24–27	361(10.8)	3.47(2.01–5.97)	<0001
27–30	218(16.9)	5.83(3.36–10.05)	<0001
30–35	90(20.3)	7.30(4.08–13.05)	<0001
>35	31(22.6)	8.40(4.31–16.35)	<0001

OR: Odds ratios; CI: confidence intervals

The high ERI level (>1) group was significantly associated with higher ORs for hyperlipidemia in both exposure to ETS and obesity groups using multiple logistic regression adjusted for gender, age, education level, income, and marital status (Table 3). For the ETS group, the ERI level >1 group had 2.22 times the risk of hyperlipidemia, which was higher than 1.39 times for the ERI<1 group. In addition, there was a higher risk for hyperlipidemia for the non-obese group (OR = 1.68) compared to the obese group (OR = 1.31). Regarding lifestyle habits, alcohol users (OR = 1.82) and betel nut chewers (OR = 1.92) had higher risks of hyperlipidemia compared to non-drinkers (OR = 1.53) and non-betel nut chewers (OR = 1.54), but there was no difference found for smokers (OR = 1.49) and non-smokers (OR = 1.58).

**Table 3:** ERI levels associated with risks of hyperlipidemia for exposure to ETS, obesity, tobacco smoking, alcohol consumption, and betel nut chewing using multiple logistic regression adjusted for gender, age, education level, income, and marital status.

Items	ERI	
	≤ 1	>1
Exposure to ETS		OR (95% CI)
No	1	1.44** (1.24–1.67)
Yes	1	2.06* (1.55–2.72)
Obesity		
No	1	1.59** (1.35–1.85)
Yes	1	1.50** (1.18–1.92)
Tobacco smoking		
No	1	1.58** (1.35–1.85)
Yes	1	1.49** (1.16–1.93)
Alcohol consumption		
No	1	1.53** (1.32–1.77)
Yes	1	1.82** (1.33–2.50)
Betel nut chewing		
No	1	1.54** (1.34–1.77)
Yes	1	1.92** (1.24–2.98)

OR: Odds ratios; CI: confidence intervals

**P<0.01

There were interaction effects of ETS exposure and ERI on risks of hyperlipidemia in the non-obese and obese groups using multiple logistic regression adjusted for gender, age, education level, income, marital status, and smoking (Table 4). For the non-obese group, those with both ETS exposure and ERI>1 had a significantly higher risk of hyperlipidemia (OR = 1.84), with a synergy index of 2.33. In the obese group, there was no significant association between risks of hyperlipidemia and having both ETS exposure and high ERI (OR = 1.62), but the synergy index of 8.45 was statistically significant. Clearly, an additive interaction of ETS exposure and high ERI on the risk of hyperlipidemia occurred in the non-obese group, showing that those with ETS exposure and perceived work stress were more vulnerable to hyperlipidemia.

**Table 4:** Synergistic effects of exposure to ETS and ERI on risks of hyperlipidemia.

		Model 1		Model 2	
		Non-obese group	Obese group	Nonsmoking group	Smoking group
ETS exposure	ERI	OR (95% CI)	OR (95% CI)	OR (95% CI)	OR (95% CI)
No	≤ 1	1	1	1	1
Yes	≤ 1	0.91 (0.67–1.22)	0.76 (0.49–1.18)	0.75 (0.54–1.05)	1.01 (0.69–1.49)
No	>1	1.46** (1.22–1.75)	1.31 (0.99–1.74)	1.41* (1.19–1.68)	1.41* (1.02–1.94)
Yes	>1	1.84** (1.44–2.37)	1.62** (1.12–2.35)	1.89** (1.47–2.42)	1.57* (1.09–2.28)
RERI		0.48(-0.39–1.35)	0.55(-0.22–1.32)	0.41(-0.39–1.22)	0.15(-0.72–1.02)
AP		0.26(-0.21–0.73)	0.34(-0.15–0.82)	0.26(-0.26–0.79)	0.10(-0.46–0.65)
Synergy index		2.33(0.16–33.95)	8.45(0.01–771847)	3.54(0.01–1548.8)	1.36(0.16–11.64)

OR: Odds ratios; CI: confidence intervals

*P<0.05

**P<0.01

Model 1: Synergistic effects of exposure to ETS and ERI on risks of hyperlipidemia for the non-obese and obese groups using multiple logistic regression adjusted for gender, age, education level, income, marital status and smoking; Model 2: Interaction of exposure to ETS and ERI on risks of hyperlipidemia for the nonsmoking and smoking groups using multiple logistic regression adjusted for gender, age, education level, income, marital status and obesity.

Table 4 shows the interaction effects of exposure to ETS and ERI on the risks of hyperlipidemia for the nonsmoker and smoker groups using multiple logistic regression adjusted for gender, age, education level, income, marital status, and obesity. There was a higher risk of hyperlipidemia for those with ETS exposure and high ERI in the nonsmoking group (OR = 1.89) and in the smoking group (OR = 1.57), with a synergy index of 3.54 and 1.36, respectively. Those with ETS exposure and high ERI were more susceptible to hyperlipidemia in the non-smoking group. Sensitivity analysis was used to evaluate the interaction effects of ETS exposure and ERI on the risks of hyperlipidemia based on three types of hyperlipidemia measurements (Table 5). There was no difference of interaction effects among the three hyperlipidemia measurement types.

**Table 5:** Sensitivity analysis for evaluating the synergistic effects of exposure to ETS and ERI on risks of hyperlipidemia based on three types of measurement.

		Self-perceived		Diagnosed by Physician		Ongoing Treatment	
		Non-obese group	Obese group	Non-obese group	Obese group	Non-obese group	Obese group
ETS exposure	ERI	OR (95%CI)	OR(95%CI)	OR (95%CI)	OR(95%CI)	OR (95%CI)	OR(95%CI)
No	≤ 1	1	1	1	1	1	1
Yes	≤ 1	1.07 (0.84–1.35)	1.02 (0.71–1.46)	0.91 (0.67–1.22)	0.76 (0.49–1.18)	0.99 (0.73–1.35)	0.84 (0.52–1.33)
No	>1	1.49** (1.28–1.74)	1.43** (1.12–1.82)	1.46** (1.22–1.75)	1.31 (0.99–1.74)	1.35** (1.11–1.64)	1.32 (0.98–1.79)
Yes	>1	1.86** (1.51–2.30)	1.53* (1.10–2.13)	1.84** (1.44–2.37)	1.62** (1.12–2.35)	1.89** (1.46–2.46)	1.77** (1.20–2.61)

## Discussion

### Risk factors for hyperlipidemia

Available data have shown that the prevalence of hyperlipidemia in Taiwan has rapidly increased due to changes in lifestyle habits and dietary behaviors, which are significant contributing factors of CVDs. Based on the second Nutrition and Health Survey for Taiwanese men from 2005 to 2008, the prevalence of hypercholesterolemia, defined as total cholesterol (TC) ≥240 mg/dL, was 13%, and the prevalence of hypertriglyceridemia, defined as triglyceride (TG) ≥ 200 mg/dL, was 21% [12]. In this study, 4.4% of civil servants who responded with self-reported hyperlipidemia were diagnosed subsequently by physicians based on high cholesterol or high triglyceride levels from annual routine medical examinations. Our results are consistent with previous studies showing that risk of hyperlipidemia were significantly associated with tobacco smoking (OR = 1.84), alcohol consumption (OR = 1.67), and betel nut chewing (OR = 2.84) [13–15]. Evidence indicated that smoking might disrupt lipid and lipoprotein metabolism, elevating plasma triglycerides, cholesterol, and low-density lipoprotein (LDL) levels and decreasing high-density lipoprotein (HDL) cholesterol level [13, 15]. Taiwan has a high rate of betel nut chewing, which stimulates one's appetite by inhibiting GABA receptors and increases the risk of obesity and occurrence of CVDs [16]. A study in Taiwan showed that the risk of obstructive coronary artery disease (CAD) was significantly associated with areca nut consumption (OR = 3.5) and showed additive interactions between areca nut use, cigarette smoking, and dyslipidemia after adjusting for other covariates [17].

Work-related stress in the workplace, including precarious work, job insecurity, overcrowding, high workload demand, inflexible operational systems, physical inactivity from extended work hours, insufficient leisure-time, unbalanced diet, depression, and anxiety, ultimately leading to CVDs [18]. Exposure to long-term stressful work can lead to high cholesterol levels, triglycerides, hematocrit, fibrinogen, and blood fluidity, and increased coagulation and platelet activity, all of which can act as “triggers” of CVDs [19, 20]. However, previous studies reported an inverse association between high levels of chronic stress and cardiovascular reactions. These studies did not consider various contributing factors of CVDs such as unhealthy lifestyle or mediating factors such as social support and obesity [21, 22]. A study for young male firefighters indicated that social support may help prevent chronic stress by enhancing recovery [23]. As hyperlipidemia is often a precursor to CVDs, the focus on it was more suitable for the bounds of this study because employees suffering from CVDs may have retired or left the workforce, and therefore, were no longer available for further analysis. Our results showed higher ERI and thus higher risk of hyperlipidemia for alcohol (OR = 1.82) and betel nut (OR = 1.92) consumers compared to non-consumption of alcohol and non-betel nuts (OR = 1.53 and OR = 1.54, respectively). However, there was an inverse relationship for smokers (OR = 1.49) and non-smokers (OR = 1.58). It is speculated that the association between the risk of hyperlipidemia and ERI may be attenuated by the effects of smoking. Notably, for alcohol drinkers and betel nut chewers, there was higher perceived work stress, which can cause a higher risk of hyperlipidemia.

### ETS, perceived work stress and hyperlipidemia and CVDs

Emerging evidence indicates a linkage among smoking and both atherosclerosis and cardiovascular disease. We hypothesize that combined exposure to high ERI and ETS can cause hyperlipidemia and increase the risk of CVDs through oxidative stress and mitochondrial damage [24]. For civil servants, ETS exposure and perceived work stress often occur together, yet their combined effects on hyperlipidemia and CVDs development currently remain unclear. Even though there is a smoke-free policy in Taiwan, civil servants may still be particularly vulnerable to ETS or THS exposure in the workplace due to their responsibility to serving citizens who smoke or are exposed to ETS. Moreover, other challenges of external competition and governmental workforce downsizing in Taiwan have led to higher perceived stress, poor sleep quality and reduced sleep hours, unbalanced diet, and physical inactivity. Consequently, civil servants have a higher risk of weight gain and hyperlipidemia due to stressful work. Also, our results exhibited interaction effects of ETS exposure and high ERI on the risk of hyperlipidemia adjusted for covariates, regardless of obesity and smoking. More critically, there were higher interaction effects that occurred in the non-obese (OR = 1.84) and non-smoking (OR = 1.89) groups, compared to the obese (OR = 1.62) and smoking (OR = 1.57) groups. The interaction effects of ETS exposure and high ERI on hyperlipidemia were dominated in the non-obese and non-smoking groups. Therefore, the adverse effects of ETS and high ERI level may explain, in part, the increased risk of hyperlipidemia, which may then cause the progression of CVDs or other metabolic diseases.

Previous studies [13, 25, 26] indicated that cigarette smoking is a contributing factor on HDL levels and functioning, revealing that each 1mg/dl decrease in plasma HDL cholesterol concentration is associated with a 2% increased risk of CVDs for men and a 3% increase for women. In a study [27] of developing countries, there was a strong positive association between ETS exposure and ischemic HD (main relative OR = 1.17–2.36) and between ETS and stroke [HR (hazard ratio) = 1.41–1.49]. Approximately 30% of heart disease (HD) risk is attributed to exposure to ETS through possible biological mechanisms related to platelet activation, endothelial dysfunction, inflammation, atherosclerosis development and progression, increased oxidative stress, decreased energy metabolism, and increased insulin resistance [28]. The mechanism of developing CVDs due to ETS exposure is linked to an increase of the proatherogenic effect of dyslipidemia. However, inconsistent findings from a study [29] reported that childhood ETS exposure was significantly associated with stroke (OR = 1.66, 95% CI = 1.29–2.13) but not CVD (OR = 1.15, 95% CI = 0.82–1.59). Limitations of the study included not adjusting for the other risk factors, retrospective self-reporting of childhood ETS exposure, and causality of exposure to diseases occurring at least 27 or more years apart [30]. Subjects with high-perceived work stress may use various negative coping strategies, such as taking drugs or using alcohol to feel better and to avoid unpleasant thoughts and painful emotions. Similarly, high levels of job strain and job demands were associated with a higher likelihood of smoking in an observational study in Finland [31]. Most of the previous studies neglected to elaborate on the combined effects of ETS exposure and perceived stress in the workplace on the risk of CVDs, metabolic diseases, and depression. Our results have indicated that the risks of hyperlipidemia were significantly associated with lifestyle habits (smoking, alcohol consumption, betel nut chewing), ERI, and weight gain for middle-aged civil servants. Moreover, both exposure to ETS and perceived stress may contribute to a synergism effect towards the risk of hyperlipidemia, especially for the non-obese and non-smoking groups.

Since the government implemented the smoke-free workplace policy in 2009 (which included federal workplaces), smokers in civil servants may have quit smoking or immediately reduced their cigarette consumption. Civil servants may have decreased ETS exposure in worksite between coworkers, but they may still have ETS exposure from citizens carrying smoke absorbed in their clothing or on their skin. Therefore, there needs to be a strict implementation of a smoke-free policy in the workplace as well as enforcement of smoking cessation and frequent remediation of THS on office walls. In addition, stressful work for civil servants should be reduced by modifying rigid bureaucratic systems, providing social support from colleagues and management, and encouraging healthy lifestyle habits.

### Strengths and limitations

Strengths of this study include using the first nationwide study of civil servants in Taiwan to examine the interaction effect of ETS exposure and perceived work stress on hyperlipidemia. The representative population was selected by PPS sampling and consistent results were validated using web- and paper-based questionnaires. Our results can be used to initiate an intervention program for high risk groups and consequently used for follow-up evaluations on the efficacy and effectiveness of the program. However, there are several limitations in the study. First, it was hard to establish causality based on the cross-sectional design. Second, complete risk factors for hyperlipidemia, such as daily diet behaviors and family history, were not collected for each participant. Although our results measured various lifestyle behaviors including cigarette smoking, betel nut chewing, sedentary time, physical activity, and sleep hours, we were unable to verify the accuracy of these self-reported behaviors. Third, the onset of hyperlipidemia was also self-reported and there could be possible recall loss from participants. Based on the sensitivity analysis for the three hyperlipidemia measurement types, our results indicated no difference on the interaction effects of ETS exposure and high ERI. In addition, our results may have a “healthy worker effect”, which can be attributed to early retirement or layoffs, and yield an underestimation in our findings. Fourth, the source and differences of workplace stress between public and private sector employees make comparing the exposures to ERI and ETS difficult and their relationship to hyperlipidemia. The limitations of this study’s population reduce the variability of these factors to produce a stronger case for the association. While the source and cause of ERI and ETS will differ between the subjects of this study (civil servants) and the general population, the findings regarding the relationship between both ETS and ERI exposure on hyperlipidemia are still applicable. Finally, the mechanisms responsible for the interaction effect of ETS exposure and perceived work stress on hyperlipidemia have not been clearly elucidated.

## Conclusion

Our results show that the risk of hyperlipidemia was significantly associated with lifestyle habits and perceived work stress. There was an interaction effect of ETS exposure and perceived work stress on hyperlipidemia for civil servants in Taiwan. Those with both exposure to ETS and perceived work stress have a higher risk of hyperlipidemia in the non-obese and non-smoking groups. Further research is needed to elucidate the biological mechanisms explaining the associations among ETS exposure, perceived work stress, weight gain, hyperlipidemia, and CVDs. It is crucial to immediately reduce ETS exposure and stressful work by enforcing smoke-free policies and various stress reduction strategies for civil servants.

## Supporting information

S1 File ERI questionnaire Chinese version. (DOCX)

## Abbreviations

ETS: Environmental tobacco smoke
CVDs: Cardiovascular diseases
HD: Health disease
BMI: Body mass index
OR: Odds ratio
ERI: Effort-reward imbalance
LDL: low-density lipoprotein
TC: total cholesterol
TG: triglyceride
HPA: Health Promotion Administration
