# Supplementary material for: Environmental tobacco smoke (ETS) and hyperlipidemia modified by perceived work stress
Source: PLoS One. 2020 Jan 16;15(1):e0227348. doi: 10.1371/journal.pone.0227348 (PMC6964979; doi:10.1371/journal.pone.0227348)
Supplement: S1 File — (DOCX) [file pone.0227348.s001.docx]

ERI Questionnaire Chinese version

**工作付出與回饋**

| 請您就目前**工作付出感受**，勾選適當選項 | **很不同意** | **不同意** | **同意** | **很同意** |
| --- | --- | --- | --- | --- |
| 1.因為工作量大，我一直有時間上的壓力。 | □ | □ | □ | □ |
| 2.工作時，我常常被打斷或受到干擾。 | □ | □ | □ | □ |
| 3.最近幾年來，我的工作負擔越來越重。 | □ | □ | □ | □ |

| 請您就目前**工作回饋感受**，勾選適當選項 | **很不同意** | **不同意** | **同意** | **很同意** |
| --- | --- | --- | --- | --- |
| 4.上司或相關人士給我應有的尊重。 | □ | □ | □ | □ |
| 5.我工作晉升機會很好。 | □ | □ | □ | □ |
| 6.我曾經歷（或預期會經歷）工作處境變壞。 | □ | □ | □ | □ |
| 7.我的工作有保障。 | □ | □ | □ | □ |
| 8.就我付出的努力與目前的成就而言，我在工作中得到應有的尊重與聲望。 | □ | □ | □ | □ |
| 9.就我付出的努力與目前的成就而言，我有相稱的工作前景。 | □ | □ | □ | □ |
| 10.就我付出的努力與目前的成就而言，我有相稱的工資收入。 | □ | □ | □ | □ |

| 請您就目前**工作投入感受**，勾選適當選項 | **很不同意** | **不同意** | **同意** | **很同意** |
| --- | --- | --- | --- | --- |
| 11.我很容易因工作上的時間壓力而煩躁。 | □ | □ | □ | □ |
| 12.我早上一起床就會開始想著工作上的事。 | □ | □ | □ | □ |
| 13.回家後我很容易就可放鬆，把工作放下。 | □ | □ | □ | □ |
| 14.熟悉我的人說，我為工作犧牲太多了。 | □ | □ | □ | □ |
| 15.我上床睡覺時還在想著工作上的事。 | □ | □ | □ | □ |
| 16.如果我沒有把今天該做的事做完，晚上我就會睡不好。 | □ | □ | □ | □ |

ERI Questionnaire English version

**Work Related Questions: Efforts and Rewards**

The following items refer to your present occupation. For each of the following statements, please indicate whether you strongly disagree, disagree, agree or strongly agree. We thank you for answering all statements.

|  | **Effort scales** | | ***Strongly disagree*** | ***Disagree*** | ***Agree*** | ***Strongly agree*** |
| --- | --- | --- | --- | --- | --- | --- |
| **ERI 1** | I have constant time pressure due to a heavy work load. | | □ | □ | □ | □ |
| **ERI 2** | I have many interruptions and disturbances while performing my job. | | □ | □ | □ | □ |
| **ERI 3** | Over the past few years, my job has become more and more demanding. | | □ | □ | □ | □ |
|  |  | |  |  |  |  |
|  | **Reward** **scales** | | ***Strongly disagree*** | ***Disagree*** | ***Agree*** | ***Strongly agree*** |
| **ERI 4** | I receive the respect I deserve from my superior or a respective relevant person. | |  |  |  |  |
| **ERI 5** | My job promotion prospects are good. | |  |  |  |  |
| **ERI 6** | I have experienced or I expect to experience an undesirable change in my work situation. | |  |  |  |  |
| **ERI 7** | My employment security is good. | |  |  |  |  |
| **ERI 8** | Considering all my efforts and achievements,  I receive the respect and prestige I deserve at work. | |  |  |  |  |
| **ERI 9** | Considering all my efforts and achievements, my job promotion prospects are adequate. | |  |  |  |  |
| **ERI 10** | Considering all my efforts and achievements, my salary / income is adequate. | |  |  |  |  |
|  |  | |  |  |  |  |
|  | **Over-commitment scales** | | ***Strongly disagree*** | ***Disagree*** | ***Agree*** | ***Strongly agree*** |
| **OC1** | I get easily overwhelmed by time pressures at work. | | □ | □ | □ | □ |
| **OC2** | As soon as I get up in the morning I start thinking about work problems. | |  |  |  |  |
| **OC3** | When I get home, I can easily relax and “switch off” work. | | □ | □ | □ | □ |
| **OC4** | | People close to me say I sacrifice too much for my job. | □ | □ | □ | □ |
| **OC5** | | Work rarely lets me go, it is still on my mind when I go to bed. | □ | □ | □ | □ |
| **OC6** | If I postpone something that I was supposed to do today, I’ll have trouble sleeping at night. | | □ | □ | □ | □ |

Form: Li J, Loerbroks A, Shang L, Wege N, Wahrendorf M, Siegrist J. Validation of a short measure of effort-reward imbalance in the workplace: evidence from China. J Occup Health. 2012;54(6):427-33. Epub 2012 Oct 12.
